# Transmission of multidrug-resistant tuberculosis in Beijing, China: An epidemiological and genomic analysis

**DOI:** 10.3389/fpubh.2022.1019198

**Published:** 2022-11-04

**Authors:** Jinfeng Yin, Hongwei Zhang, Zhidong Gao, Hui Jiang, Liyi Qin, Chendi Zhu, Qian Gao, Xiaoxin He, Weimin Li

**Affiliations:** ^1^Beijing Chest Hospital, Capital Medical University, Beijing, China; ^2^National Tuberculosis Clinical Laboratory, Beijing Tuberculosis and Thoracic Tumor Research Institute, Beijing, China; ^3^Tuberculosis Prevention and Control Institute, Beijing Center for Disease Prevention and Control, Beijing, China; ^4^Key Laboratory of Medical Molecular Virology, School of Basic Medical Sciences, Fudan University, Shanghai, China

**Keywords:** transmission, multidrug-resistant tuberculosis, whole-genome sequencing, migrants, epidemiology

## Abstract

**Background:**

Understanding multidrug-resistant tuberculosis (MDR-TB) transmission patterns is crucial for controlling the disease. We aimed to identify high-risk populations and geographic settings of MDR-TB transmission.

**Methods:**

We conducted a population-based retrospective study of MDR-TB patients in Beijing from 2018 to 2020, and assessed MDR-TB recent transmission using whole-genome sequencing of isolates. Geospatial analysis was conducted with kernel density estimation. We combined TransPhylo software with epidemiological investigation data to construct transmission networks. Logistic regression analysis was utilized to identify risk factors for recent transmission.

**Results:**

We included 241 MDR-TB patients, of which 146 (60.58%) were available for genomic analysis. Drug resistance prediction showed that resistance to fluoroquinolones (FQs) was as high as 39.74% among new cases. 36 (24.66%) of the 146 MDR strains were grouped into 12 genome clusters, suggesting recent transmission of MDR strains. 44.82% (13/29) of the clustered patients lived in the same residential community, adjacent residential community or the same street as other cases. The inferred transmission chain found a total of 6 transmission events in 3 clusters; of these, 4 transmission events occurred in residential areas and nearby public places. Logistic regression analysis revealed that being aged 25–34 years-old was a risk factor for recent transmission.

**Conclusions:**

The recent transmission of MDR-TB in Beijing is severe, and residential areas are common sites of transmission; high levels of FQs drug resistance suggest that FQs should be used with caution unless resistance can be ruled out by laboratory testing.

## Introduction

Multidrug-resistant tuberculosis (MDR-TB) poses a serious threat to global tuberculosis control programs. According to the World Health Organization (WHO) report, there were an estimated 360,000 MDR-TB cases in 2020, but only 38% received treatment (1). MDR-TB is associated with a lower cure rates (15% vs. 4%), higher mortality (59% vs. 85%) and high treatment cost (5,659$/person) compared with drug-susceptible TB (1). China ranks second among the 27 countries most burdened by TB worldwide, contributing to 14% of all estimated MDR-TB cases (1). MDR-TB is thus a global problem and a major public health issue in China. Recent transmission is primarily responsible for driving the global endemic of MDR-TB (2–4). Therefore, understanding MDR-TB transmission patterns is crucial to informing public health efforts in carrying out effective interventions.

With the development of whole-genome sequencing technology, we can define a threshold for the number of genomic differences above which direct transmission is unlikely (5). This approach has the advantage of providing a simple way for judging transmission occurrence but cannot reconstruct an accurate transmission network. Most reconstructions rely heavily upon fieldwork data. However, due to the lack of investigation data and participant recall bias, identifying specific and accurate person-to-person transmission events is challenging. Nevertheless, it is possible to infer transmission from sequencing data using alternative approaches; several such methods have been proposed (6–8). Phylogenetic networks based on whole-genome sequencing can be used to identify putative source cases, super-spreaders, and transmission directions in the absence of comprehensive epidemiological data. Therefore, there has been increasing integration of genetic and epidemiological data to construct a more accurate transmission network and infer transmission dynamics (9).

Beijing is one of the most populous cities in the world with a resident population of 21.9 million. The city hosts large numbers of migrants from other parts of China, who account for around 38.5% of its population (10). Compared with other Chinese regions, the incidence of TB is relatively low, but slowly declining (11). Internal migrants are at a higher risk of TB (9, 12) and may spread the disease during their travels.

Understanding TB transmission is of great importance for identifying the origins of the disease and the population that is at risk of infection (13). However, the mechanisms involved in MDR-TB transmission in Beijing have not been investigated to date. We combined epidemiological, molecular genetics, and spatial analysis to investigate the transmission dynamics of MDR-TB in Beijing, China. We quantified MDR-TB recent transmission, and identified risk factors for transmission and high-risk geographic sites. Our findings provide a scientific basis for public health agencies to create more effective strategies for TB control.

## Methods

### Study design and population

Beijing is the capital of China and is divided into 16 districts. All individuals with suspected TB are referred to local designated hospitals for diagnosis and treatment. Drug resistance screening by GeneXpert technology is performed in all patients diagnosed with etiologically positive TB. All cases in Beijing confirmed to harbor rifampicin-resistant TB (RR-TB) are registered in the TB management information system. Sputum samples from these cases are collected for culture and drug susceptibility testing with rifampin and isoniazid to further identify patients with MDR-TB. All culture-positive strains are mainly stored in two designated hospitals (Beijing Chest Hospital and Beijing Institute of Tuberculosis Control). This observational study included all cases with culture-confirmed MDR-TB who were reported by the Beijing Chest Hospital during 2018–2020.

### Whole-genome sequencing

Strains of MDR-TB were re-grown on Löwenstein-Jensen medium and their genomic DNA was extracted using the cetyl trimethyl ammonium bromide method (14). For each sample, a pair-end library was constructed and sequenced on an Illumina platform (Illumina), with an expected 250× coverage. Paired-end reads were mapped to the reference genome H37Rv (GenBank AL123456) with Bowtie2. The SAMtools/VarScan suite was used for SNP calling with a mapping quality > 30. Fixed SNPs (frequency ≥ 75%), excluding those present in genes associated with drug-resistance and repetitive regions of the genome (e.g., PPE/PE-PGRS family genes, phage sequence, insertion or mobile genetic elements), were used to calculate the pairwise SNP distances. A genomic cluster was defined as strains with a genetic distance of ≤ 12 SNPs, suggesting they were the result of a recent transmission (4). Strains that differed by >12 SNPs as compared with all other strains were defined as unique strains. The strains were classified into different lineages according to Coll et al. (15). L2 strains were classified into L2.1, L2.2 and L2.3 (16). The L2.3 represents “modern” Beijing and others are “ancient” Beijing. The drug-resistance profile was predicted for 14 anti-TB drugs based on the mutations reported to be associated with resistance (17). Phylogenetic trees were constructed using MEGA (version 7.0). Visualization of the bacteriological information was performed by using the Interactive Tree Of Life (https://itol.embl.de/). The sequencing data have been deposited with links to BioProject accession number PRJNA888557 in the National Center for Biotechnology.

Information BioProject database (https://www.ncbi.nlm.nih.gov/bioproject/).

### Epidemiological investigation

Consenting participants underwent a structured interview. Based on self-administered questionnaires, we collected data on close contacts, social contacts, and places they frequented in the 5 years preceding their MDR-TB diagnosis, including the detailed addresses of their residences, workplaces, and entertainment venues. In addition, we investigated changes in behavior following their diagnosis. Individuals with confirmed epidemiological links were defined as patients knew each other. Individuals with probable epidemiological links were defined as: cohabitating at the same address or complex or shared locations where transmission likely occurred, including in a neighborhood complex or street in the same district.

### Statistical analysis

Participant demographic and clinical characteristics were collected from the TB management information system and the Beijing Chest Hospital clinical system. Statistical analyses were performed in R (version 4.1.1). The Chi-square test and Fisher's exact tests were used to compare differences between a cluster group and a unique group. Variables with *p*-value ≤ 0.5 on Chi-square test were entered into a multivariate logistic regression, which was used to assess possible associations of genomic clustering and estimate odds ratios (ORs) and 95% confidence intervals (CIs). Significance was determined by *p* < 0.05. Spatial analysis and visualization were performed in ArcGIS (version 10.2). We used kernel density estimation methods with Gaussian smoothing to analyze the aggregation of patients with MDR-TB.

We inferred transmission chains in clustered patients based on whole genome sequencing data. This study only inferred the transmission relationship for cluster size ≥ 4. We first used BEAST (version 2.6.6) to infer a timed phylogeny tree with the genomic sequencing data. This timed phylogeny tree was then used as input for the transmission tree inference using TransPhylo (https://github.com/xavierdidelot/TransPhylo) R package. We also predicted transmission probability based on the model (Supplementary materials).

## Results

### Study population and characteristics

**Figure 1:**
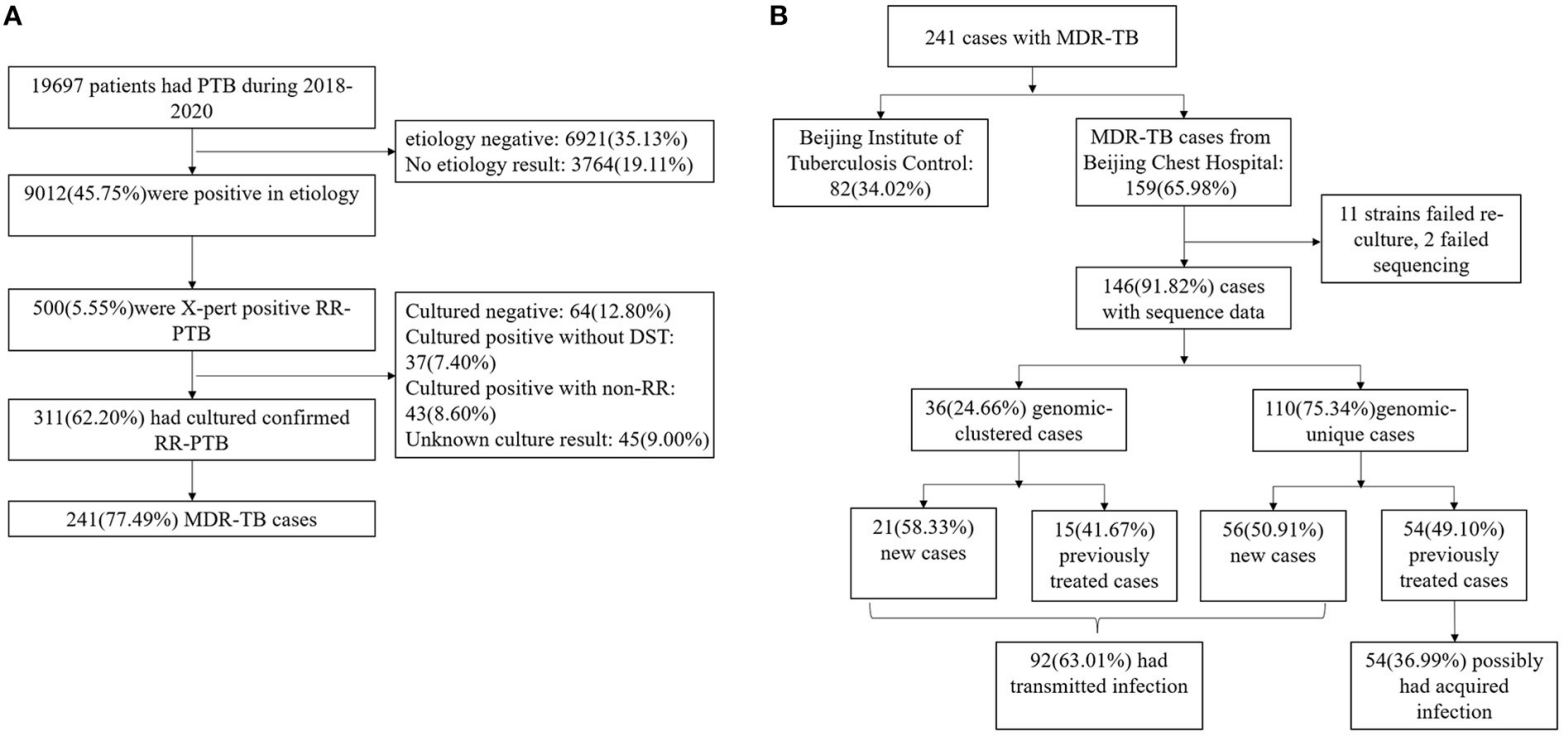
**(A)** Sample enrollment of MDR-TB. **(B)** Classification of MDR-TB based on treatment history and genomic analysis.

9,012 individuals tested etiology-positive for Mycobacterium Tuberculosis (MTB) during 2018–2020, and 500 (5.55%) of these patients were classified as RR-TB by GeneXpert. After excluding culture-negative (12.80%), culture-positive non-RR-TB (8.6%), and missing culture or drug susceptibility (16.40%) cases, 311 (62.20%) individuals were confirmed to have RR-TB by phenotypic drug susceptibility testing; of these, 241 (77.49%) were MDR-TB. 159 (65.98%) MDR-TB cases were diagnosed and treated in the Beijing Chest Hospital and 146 (60.58%) had a clinical isolate suitable for analysis (Figure 1). The 146 individuals were representative of the whole diagnosed MDR-TB cohort in terms of age, sex, and internal migration status (defined as those who were not born in Beijing; home provinces were be inferred from the national identification number) (Supplementary Table 1).

The general characteristics of MDR-TB cases are summarized in Table 1. Among the 146 participants, 104 (71.23%) were male, with a median age of 44 years [interquartile range (IQR), 28–56 years]. 17.12% (25/146) were retired and 16.44% (24/146) were unemployed. New cases accounted for 52.74% (77/146) of all cases. 57(39.04%) patients were classed as internal migrants. Internal migrants with MDR-TB (31[27–43]) were significantly younger than MDR-TB local residents (53[36–59]) (*p* < 0.001). 50.88% (29/57) of internal migrants had resided in Beijing for <5 years (determined based on residence time at the address provided at the time of diagnosis). A greater proportion of migrants worked in retail (17.54% vs. 2.25%; *p* < 0.001).

**Table 1:** Demographic, clinical, and bacteriological characteristics of internal migrant patients and resident patients diagnosed in Beijing.

	**Total** **(*n* = 146)**	**Migrant patients** **(*n* = 57)**	**Resident patients** **(*n* = 89)**	***P*-value**
Sex				0.980
Male	104 (71.23%)	41 (70.93%)	63 (70.79%)	
Female	42 (28.77%)	16 (28.07%)	26 (29.21%)	
Age group				< 0.001
15–24	15 (10.27%)	9 (15.79%)	6 (6.74%)	
25–34	40 (27.40%)	26 (45.61%)	14 (15.73%)	
35–44	24 (16.44%)	9 (15.79%)	15 (16.85%)	
45–54	19 (13.01%)	6 (10.53%)	13 (14.61%)	
≥55	38 (26.03%)	7 (12.28%)	41 (46.07%)	
Residence years				< 0.001
< 5	29 (19.86%)	29 (50.88%)	0 (0.00%)	
5–10	20 (13.70%)	20 (35.09%)	0 (0.00%)	
≥10	97 (66.44%)	8 (14.04%)	89 (100.00%)	
Occupation				< 0.001
Farmers	17 (11.64%)	5 (8.77%)	12 (13.48%)	
Workers	13 (8.90%)	3 (5.26%)	10 (11.24%)	
Retired people	24 (16.44%)	3 (5.26%)	21 (23.60%)	
Unemployed	25 (17.12%)	8 (14.04%)	17 (19.10%)	
Retail workers	12 (8.22%)	10 (17.54%)	2 (2.25%)	
Others	55 (37.67%)	28 (49.12%)	27 (30.34%)	
TB history*				0.486
New case	77 (52.74%)	33 (57.89%)	45 (50.56%)	
Previous treatment	69 (47.26%)	24 (42.11%)	44 (49.44%)	
Cough				0.259
Yes	87/116 (75.00%)	33/40 (82.50%)	54/76 (71.05%)	
No	29/116 (25.00%)	7/40 (17.50%)	22/76 (28.95%)	
Smear status				0.525
Positive	98 (67.12%)	36 (63.16%)	62 (69.66%)	
Negative	48 (32.88%)	21 (36.84%)	27 (30.34%)	
Putative compensatory mutation in rpoA, rpoB and rpoC				0.079
Yes	43 (29.45%)	22 (38.60%)	21 (23.60%)	
No	103 (70.55%)	35 (61.40%)	68 (76.40%)	
Genomic clustered				0.029
Yes	36 (24.65%)	8 (14.04%)	28 (31.46%)	
No	110 (75.34%)	49 (85.96%)	61 (68.54%)	

^*^Data from case management system of Beijing Chest Hospital.

### Whole-genome sequencing analysis

**Figure 2:**
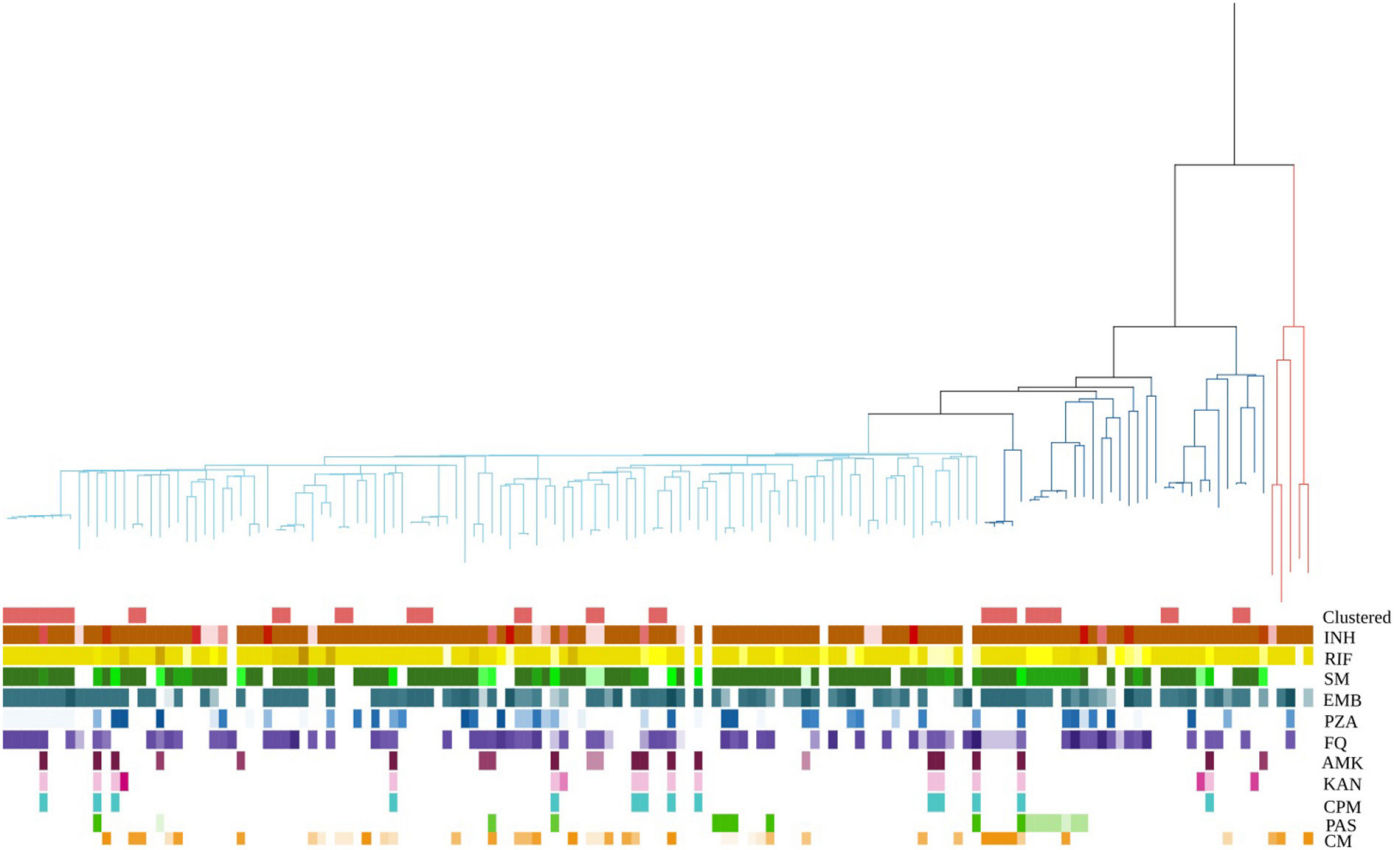
MDR-MTBC phylogeny and resistance mutations of isolates. First row showed whether it is clustered. Others show drug resistance associated mutations to first-line and second-line drugs (different mutations represented by different colors), and putative compensatory mutations in the RNA polymerase genes rpoA, rpoB and rpoC. MTBC lineage are differentiated into three clades. Red, blue, and cyan branches indicated lineage 4, ancient Beijing, and modern Beijing strains, respectively. INH, isoniazid; RIF, rifampicin; SM, streptomycin; EMB, ethambutol; Z PZA, pyrazinamide; FQ, fluoroquinolone; AMK, amikacin; KAN, kanamycin; CPM, capreomycin; PAS, para aminosalicylic acid; CM, compensatory mutations.

A maximum likelihood phylogeny tree was constructed for the 146 MDR-TB strains (Figure 2). The average SNP pairwise distances between strains were 358 ± 243 SNPs (Supplementary Figure 1). Most of the MTB isolates belonged to lineage 2.3 (*n* = 111; 76.03%), followed by lineage 2.2 (*n* = 30; 20.55%), and lineage 4 (*n* = 5; 3.42%). The tree also displays the resistance profile for 11 anti-TB drugs based on the presence of validated resistance-conferring mutations (Supplementary Table 2). 54.79% (80/146) were classified as fluoroquinolones (FQs) resistant; among new cases of MDR-TB, the resistance ratio to any FQs was 39.74%. Considering that FQs resistance may be caused by the use of FQs between the time of diagnosis by GeneXpert and the sampling time of culture-positive strains, we descripted the sampling time distribution of the strains (Supplementary Figure 2). We found that the average time interval between diagnosis and sampling was 24 days. After excluding 21 (14.38%) cases with a delay > 30 days, the proportion of new cases harboring FQs resistance mutations was 40.29%, indicating that we could assume that any bias associated with sampling delay was minimal and could be disregarded. In addition, 43(29.45%) strains had compensatory mutations in rpoA, rpoB, or rpoC genes; rpoC V483G and rpoC V483A were the most frequent mutations (Supplementary Figure 3).

36 (24.66%) strains were grouped into 12 genome clusters, whose group sizes ranged from two to eight cases, suggesting recent transmission of MDR strains. The presence of MDR-TB among new cases suggested transmission of MDR strains. If the cases of MDR-TB among new cases were combined with those in the genomic clusters, 63.01% (92/146) of cases were likely caused by the transmission of MDR strains (Figure 1B).

### Geographic distribution of MDR-TB

**Figure 3:**
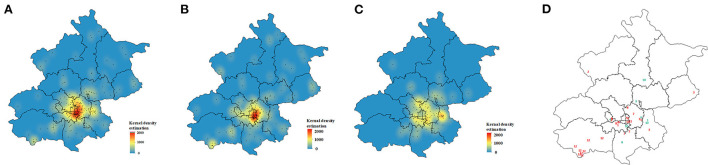
Spatial distribution of multidrug-resistant tuberculosis cases. **(A)** Kernel density estimation for all 241 patients, **(B)** resident patients and **(C)** migrant patients. **(D)** Spatial distribution of genetic clusters; red numbers represent resident patients and green numbers represent migrant patients.

The MDR-TB patient geographic distribution was heterogeneous. The majority of cases aggregated in central urban areas; Chaoyang District was the source of the largest number of cases, followed by Fengtai and Tongzhou Districts. Kernel density analysis of residents and internal migrants showed two distinct spatial distributions. Resident cases aggregated in central urban areas, while internal migrants were dispersed distributed around the urban area (Figure 3).

Of the 12 clusters, 7 were resident-only clusters and 2 were migrant-only clusters. 3 mixed clusters indicated that MDR-TB transmission had occurred between residents and migrants. We noted spatial aggregation within most resident-only clusters (3, 5, 6, 11, and 12) with hot spots in downtown and suburban areas. Individuals in migrant-only clusters (9, 10) and mixed clusters (1, 8), except for cluster 4 tended to reside in different districts (Figure 3D).

### Epidemiologic survey of the study population

Of the 146 cases, there were nine deaths and 84 (57.53%) agreed to be interviewed by telephone or in person. We managed to establish probable epidemiological links for 15 cases, before their MDR-TB diagnosis. 9 individuals lived in the same community as other cases, and 15 had utilized the same entertainment venues, such as restaurants and supermarkets. Only 4 patients reported confirmed epidemiological links with others (all are friends). After the diagnosis of MDR-TB, 22.62% (19/84) continued to frequent public places and 9 were ≥ 55 years old. In the personal habit survey, 44.44% (24/54) stated that they would not cover their mouth during coughing and sneezing, and 16.32% (8/49) said they would expectorate sputum when necessary.

### Inferring transmission chains of clustered cases

Overall, 29 (80.55%) of 36 individuals completed the survey, and seven refused or were lost to follow-up. Confirmed or probable epidemiological links were identified in 13 (44.82%) of 29 cases. 13 (44.82%) lived in the same or adjacent residential community, or on the same neighborhood street (distance ≤ 3 km), or shared public facilities such as restaurants. Eight of the clustered patients were internal migrants. Two cases came from the same county where the transmission likely occurred. Other migrants traveled to Beijing from different provinces, suggesting that local transmission likely occurred after arrival in Beijing (Supplementary Table 3).

**Figure 4:**
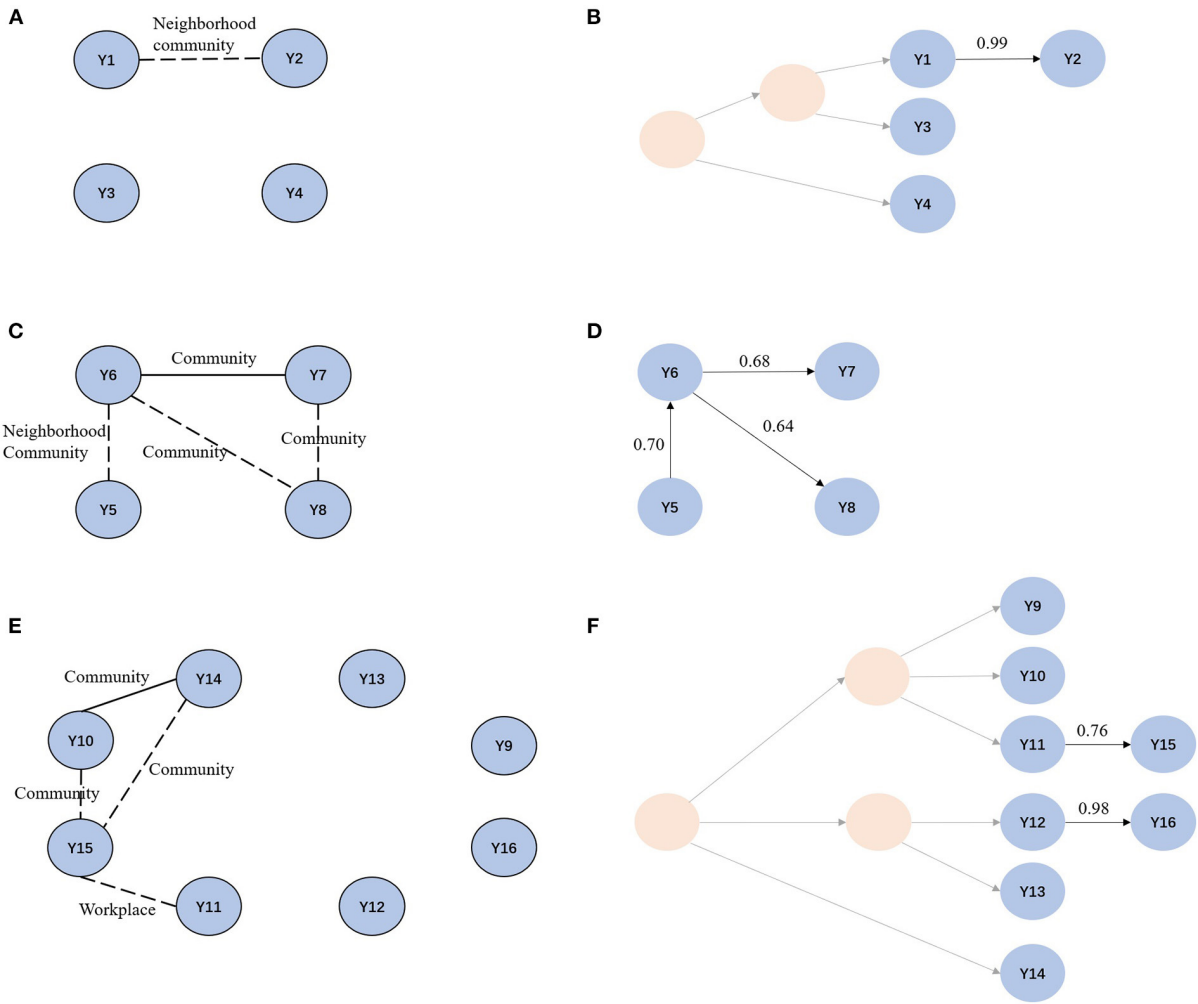
Social network and transmission chain for cluster 3, cluster 4 and cluster 12. **(A, C, E)** Social network for cluster 3, cluster 4, and cluster 12. Solid line indicates confirmed epidemiological links. Dotted line indicates probable epidemiological links. **(B, D, F)** Transmission chain for cluster 3, cluster 4 and cluster 12. Light yellow circles represent missing samples.

We then focused on clusters 3, 4, and 12, each of which had a cluster size of ≥4, using TransPhylo. In cluster 3, Y1 and Y2 lived in neighborhood communities, and often attended the same restaurants near their homes. We inferred that Y2 was a secondary case to Y1, with a transmission probability of 0.99. Individuals in cluster 4 lived in the neighborhood or the same communities, and Y6 and Y7 frequented restaurants near their homes. We found that Y5 first infected Y6, and Y6 further infected Y8 and his friend Y7. The probabilities of direct transmissions were > 0.6. The transmission chain provided supporting evidence for the epidemiological data. Cluster 12 included 8 cases; three lived in the same village and frequented the same restaurants. In addition, Y11 and Y15 worked on the same street, approximately 3.3 km apart. In the transmission chain of cluster 12, we confirmed the transmission relationship between Y11 and Y15. We also identified a casual transmission event between Y12 and Y16 without any apparent epidemiological link. Markedly, although there was an epidemiological link between Y10 and Y14, we found no direct transmission relationship, likely due to missing intermediary cases (Figure 4).

### Risk factors for recent transmission

We compared groups to identify the risk factors associated with the recent transmission of MDR-TB. The proportion of female was significantly higher among clustered cases than in unique cases (44.44% vs. 23.64%; *p* = 0.029), and residents had a higher proportion than internal migrants (31.04% vs. 14.64%; *p* = 0.029) (Supplementary Table 4). In the multivariate logistic analysis, the age group 25–34 had the highest risk for recent transmission (OR, 3.19; 95% CI, 1.14–9.35). As the number of cases was small, we did not identify any other significant factors (Table 2).

**Table 2:** Multivariable logistic regression on the risk factors of multidrug resistance clustering.

	**Total**	**Multivariable OR (95% CI)**	***p*-value**
Sex			
Male	104 (71.23%)	Ref	–
Female	42 (28.77%)	2.19 (0.90–5.32)	0.081
Age group			
25–34	40 (27.40%)	3.19 (1.14–9.35)	0.028
others	106 (72.60%)	Ref	–
Residence years			
< 10	49 (33.56%)	Ref	–
≥10	97 (66.44%)	3.38 (0.738–23.23)	0.225
Birth in Beijing	89 (60.95%)	1.53 (0.28–12.21)	0.643
Farmers	17 (11.64%)	2.50 (0.75–8.11)	0.126
Smear positive	98 (67.12%)	1.95 (0.76–5.43)	0.179

## Discussion

We combined whole-genome sequencing, geospatial analysis, and epidemiological investigation to characterize the transmission of MDR-TB during a 3-year interval in Beijing, China. We inferred that approximately 63% of MDR-TB cases were due to transmission. 44.82% of the clustered patients were co-infected with others in residential communities and related public facilities. Based on reconstructed transmission networks, we identified 6 transmission events in three clusters; of these, four transmission events occurred in and near residential areas.

24.66% of our MDR-TB cases were attributable to recent transmission, a higher proportion than that reported in low-burden countries such as the US (18), but with little difference from Chinese regions such as Shenzhen (19) (25%) and Shanghai (4) (32%). The WHO pointed out that the detection rate of MDR-TB in China (27%) was much lower than the global average (44%) (1), suggesting that many cases are undetected and treated. This further implies that the transmission of MDR-TB is likely more extensive than seen in the present study.

Fluoroquinolones are one of the key antituberculosis drugs for MDR/RR-TB treatment (20). We found resistance to any FQs of up to 40% among new MDR-TB cases in our study. The proportion was higher than the national average (1, 21). But others have reported an even higher resistance to the FQs drug moxifloxacin at 53.9%, in the eastern region of China (22). FQs are broad-spectrum antibiotics and one of the most commonly prescribed classes of antibiotics in China, patients may be exposed to FQs to treat other microbial infections leading to resistance acquisition. It has been reported that Beijing is one of the largest regions for antibiotic use in China (23), which creates a greater risk for resistance to FQs in TB patients in this region. However, in most areas of China, including Beijing, drug susceptibility testing is only performed for parts of FQs drugs (22). Therefore, we caution that the use of FQs drugs as part of second-line regimens should be approached with caution, and FQs drugs susceptibility testing, especially molecular drug susceptibility testing, should be recommended in the national tuberculosis guidelines.

Internal migrants are thought to play an important role in TB transmission (24–26). For example, TB in the Baoan District of Shenzhen principally stems from the reactivation of infections acquired by migrants in their home provinces (27). However, our results showed that the clustering rate among local residents was higher than among internal migrants, suggesting that the former played a major role in MDR-TB transmission, especially in the central urban area of Beijing. We speculated that this is attributable to local living conditions and the structure of the population. First, the central urban area had a higher population density and housing costs than the surrounding areas (10), resulting in a large crowded living environment, that likely contributed to recent transmission. In addition, most residents in the central area were middle-aged and elderly, and had more limited social circles and places of recreation; this may explain why recent transmission primarily occurred among residents of the central area. Compared with the spatial aggregation of resident-only clusters in urban centers, we found more dispersed spatial patterns for migrant-only and mixed clusters, which means that transmission events could occur through more casual contact in some settings, such as on public transportation, or in entertainment venues and hospitals (28).

Close household contact was a significant risk factor for TB transmission, but most TB cases were not attributable to household contacts in high-incidence settings (29). None of the patients in this study were in close household contact with TB patients. However, 44.82% of the patients lived in the same or adjacent residential community or street with other individuals. In addition, we identified six transmission events by reconstructing transmission networks; four occurred in and near residential areas. This suggested that residential areas and nearby public places were vitally important for TB transmission. A study of TB in Shanghai reached similar conclusions that transmission events were observed in game rooms near residential areas (4). As such, we need to place a greater emphasis on residential communities as potential areas of TB transmission, because the majority of individuals exposed to small risks can account for more cases than a few individuals exposed to large risks. However, residential communities, including supermarkets, restaurants, entertainment facilities, etc., are locations in which the implementation of prevention and control measures is logistically difficult if not impossible. Therefore, the need for further research on the characteristics of TB transmission in residential communities remains.

Patient management is an important component of disease control and prevention because the probability of transmission between a susceptible and an infectious person is significantly influenced by the behaviors of the infected individual. For example, covering of the mouth and nose during breathing, coughing, or sneezing can reduce TB transmission in gathering places (30). However, in our survey, 44% of our cohort reported inappropriate behaviors such as coughing without covering their mouths and spitting in public. These behaviors and relatively lax self-isolation protocols can increase the risk of infection of uninfected people. Therefore, we need to improve the education of patients and the wider public to help control transmission.

Our study has some limitations. First, our ability to infer MDR-TB transmission patterns in Beijing depended on how complete our sampling of MTB isolates was during the study. Not all registered cases were culture-positive and we only included 60% of MDR-TB patients in Beijing. The absence of some cases might have masked some transmission events and epidemiological relationships. Second, the retrospective study design and limited timeframe made it difficult to fully cover all MDR-TB cases and strains in the population. Third, we might have missed cases among migrants who returned to their home province for treatment, potentially leading to an underestimation of the role of the migrants in disease transmission.

Taken together, the transmission of MDR-TB played an important role in the burden of MDR-TB. We found that residential areas were common transmission sites, and further work is needed to properly understand the characteristics of TB transmission in community settings. In a setting with high-level FQs drug resistance in MDR-TB patients, FQs should be used with caution when treating drug-resistant patients unless susceptibility can be confirmed by molecular or phenotypic methods, to reduce the burden of MDR-TB.

## Data availability statement

The datasets presented in this study can be found in the National Center for Biotechnology Information BioProject Database (https://www.ncbi.nlm.nih.gov/bioproject/), accession number PRJNA888557.

## Ethics statement

The studies involving human participants were reviewed and approved by the Ethical Review Board of Beijing Chest Hospital (No. LW2022-005). The patients/participants provided their written informed consent to participate in this study.

## Author contributions

WL and XH conceived, designed, and supervised the study. XH, ZG, and HZ collected the data. JY and LQ cleaned the data. JY and CZ analyzed the data. JY wrote the drafts of the manuscript. WL, HJ, and QG interpreted the findings, commented, and revised the drafts of the manuscript. All authors read and approved the final manuscript.

## Funding

This study was funded by grants from the National Natural Science Foundation of China (U1903118).

## Conflict of interest

The authors declare that the research was conducted in the absence of any commercial or financial relationships that could be construed as a potential conflict of interest.

## Publisher's note

All claims expressed in this article are solely those of the authors and do not necessarily represent those of their affiliated organizations, or those of the publisher, the editors and the reviewers. Any product that may be evaluated in this article, or claim that may be made by its manufacturer, is not guaranteed or endorsed by the publisher.
