# Supplementary material for: Transmission of multidrug-resistant tuberculosis in Beijing, China: An epidemiological and genomic analysis
Source: Front Public Health. 2022 Nov 4;10:1019198. doi: 10.3389/fpubh.2022.1019198 (PMC9672842; doi:10.3389/fpubh.2022.1019198)
Supplement: Supplementary file 1 [file Data_Sheet_1.docx]

**Supplementary Text**

**Methods**

**Transmission inference based on timed phylogeny trees and a Bayesian-based approach**

We first used BEAST (version 2.6.6) to infer a timed phylogeny tree with the genomic sequencing data, with a model of a coalescent constant population size with an initial prior value of 1.5 and strict molecular clock rate. The input XML file was modified to specify the number of invariant sites. The model was run using a Markov Chain Monte Carlo (MCMC) chain length of 10,000,000 with 10% burn-in and sampling very 1,000 interactions. A Maximum clade credibility tree was generated using TreeAnnotator (version 1.10.4), with 10% burn-in and visualized and transformed using FigTree (version 1.4.4). This timed phylogeny tree was then used as input for the transmission tree inference using a R package called TransPhylo (<https://github.com/xavierdidelot/TransPhylo>). The model was run a MCMC length of 1,000,000.

Supplement Table 1: Characteristics of All Multidrug-resistant Tuberculosis, with Whole-Genome Sequencing Results, MDR diagnosed by Beijing Institute of Tuberculosis Control and MDR diagnosed by Beijing Chest Hospital.

|  | All cases (n=241) | Cases with WGS (n=146) | P-value | Beijing Institute of Tuberculosis Control (n=82) | Beijing Chest Hospital (n=159) | P-value |
| --- | --- | --- | --- | --- | --- | --- |
| Sex |  |  | 0.476 |  |  | 0.076 |
| Male | 162 (67.22%) | 104 (71.23%) |  | 49 (59.76%) | 113 (71.07%) |  |
| Female | 79 (32.78%) | 42 (28.77%) |  | 33 (40.24%) | 46 (28.93%) |  |
| Age |  |  | 0.956 |  |  | 0.476 |
| 15-24 | 23 (9.54%) | 15 (10.27%) |  | 6 (7.32%) | 17 (10.69%) |  |
| 25-34 | 67 (27.80%) | 40 (27.40%) |  | 24 (29.27%) | 43 (27.04%) |  |
| 35-44 | 43 (17.84%) | 24 (16.44%) |  | 17 (20.73%) | 26 (16.35%) |  |
| 45-54 | 36 (14.94%) | 19 (13.01%) |  | 15 (18.29%) | 21 (13.21%) |  |
| ≥55 | 72 (29.88%) | 48 (32.88%) |  | 20 (24.39%) | 52 (32.70%) |  |
| Birth in Beijing |  |  | 0.542 |  |  | 0.417 |
| Yes | 138 (57.26%) | 89 (60.95%) |  | 44 (53.66%) | 94 (59.12%) |  |
| No | 103 (42.74%) | 57 (39.04%) |  | 38 (46.34%) | 65 (40.88%) |  |
| Living district |  |  | 0.366 |  |  | <0.05 |
| Chaoyang | 41 (17.01%) | 32 (21.92%) |  | 7 (8.54%) | 34 (21.38%) |  |
| Tongzhou | 27 (11.20%) | 22 (15.07%) |  | 2 (2.44%) | 25 (15.72%) |  |
| Fengtai | 30 (12.45%) | 19 (13.01%) |  | 9 (10.98%) | 21 (13.21%) |  |
| Fangshan | 21 (8.71%) | 17 (11.64%) |  | 4 (4.88%) | 17 (10.69%) |  |
| Daxing | 19 (7.88%) | 10 (6.85%) |  | 8 (9.76%) | 11 (6.92%) |  |
| Shunyi | 18 (7.47%) | 9 (6.16%) |  | 9 (10.98%) | 9 (5.66%) |  |
| Haidian | 25 (10.37%) | 5 (3.42%) |  | 18 (21.95%) | 7 (4.40%) |  |
| Changping | 12 (4.98%) | 5 (3.42%) |  | 5 (6.10%) | 7 (4.40%) |  |
| Others | 39 (16.18%) | 18 (12.33%) |  | 20 (24.39%) | 28 (17.61%) |  |

Supplement Table 2: Resistance profile of MDR-TB cases in Beijing, stratified by treatment history.

|  | Total (n=146) | New cases (n=78) | Previous treated  (n=68) |
| --- | --- | --- | --- |
| First-line drug resistance | |  |  |
| INH | 141 (96.58%) | 76 (97.43%) | 65 (95.58%) |
| RIF | 142 (97.26%) | 76 (97.43%) | 66 (97.05%) |
| SM | 118 (80.82%) | 65 (83.33%) | 53 (77.94%) |
| EMB | 107 (73.29%) | 55 (70.51%) | 52 (76.47%) |
| PZA | 63 (43.15%) | 28 (35.89%) | 35 (51.47%) |
| Second-line drug resistance | |  |  |
| FQ | 80 (54.79%) | 31 (39.74%) | 49 (72.05%) |
| AMK | 22 (15.07%) | 7 (8.97%) | 15 (22.05%) |
| KAN | 18 (12.33%) | 6 (7.69%) | 12 (17.64%) |
| CPM | 14 (9.59%) | 4 (5.12%) | 10 (14.70%) |
| PAS | 17 (11.64%) | 5 (6.41%) | 12 (17.64%) |
| ETO | 22 (15.07%) | 5 (6.41%) | 17 (25.00%) |

Supplement Table 3 Characteristics of multidrug-resistant tuberculosis genomic clusters based on whole-genome sequencing analysis

|  | cluster size | no. male | average age | number of new cases | number of patients living in the same district | average pairwise geographic distance (Km) | no. migrant patients | no. patients receiving investigation | risk factors | known social link | epidemiological links |
| --- | --- | --- | --- | --- | --- | --- | --- | --- | --- | --- | --- |
| Cluster01 | 2 | 1 | 43.5 | 2 | 0 | 34 | 1 | 2 | Physician (1); | Unknown | Unknown |
| Cluster02 | 2 | 2 | 37.5 | 1 | 0 | 83 | 0 | 1 | Diabetes (1) | Unknown | Unknown |
| Cluster03 | 4 | 3 | 55 | 3 | 2 | 41 | 0 | 3 | Imprisonment (1);  diabetes (2) | Neighborhood community(2;2.6km);  restaurant (2) | Probable |
| Cluster04 | 4 | 2 | 29 | 1 | 4 | 2.2 | 1 | 2 | ND | Friend (2); community(4;2.2km);  restaurant (2) | Yes |
| Cluster05 | 2 | 2 | 55 | 1 | 2 | 2.7 | 0 | 2 | Chess room (1);  diabetes (1) | Neighborhood street (2;2.7km） | Probable |
| Cluster06 | 2 | 2 | 58 | 1 | 2 | 3.5 | 0 | 1 | Diabetes (1) | Neighborhood street（2;3km） | Probable |
| Cluster07 | 2 | 1 | 62.5 | 2 | 0 | 25 | 0 | 2 | Chess room (1);  diabetes (1) | Unknown | Unknown |
| Cluster08 | 3 | 1 | 32 | 2 | 0 | 23 | 1 | 2 | Hospitalization (1); diabetes (1) | Unknown | Unknown |
| Cluster09 | 2 | 0 | 27.5 | 2 | 0 | 21 | 2 | 2 | ND | Source county (2) | Probable |
| Cluster10 | 3 | 1 | 25 | 2 | 0 | 50 | 3 | 2 | ND | Unknown | Unknown |
| Cluster11 | 2 | 1 | 46.5 | 0 | 0 | 6.3 | 0 | 2 | ND | Unknown | Unknown |
| Cluster12 | 8 | 4 | 53.5 | 3 | 8 | 8.4 | 0 | 8 | Chess room (1); diabetes (4) | Friend (2); community(3;2.5km); restaurant (3) | Probable |

Supplement Table 4: Demographic, Clinical, and Bacteriological Characteristics of Genomic-Clustered and Unique Cases of Multidrug-resistant Tuberculosis in Beijing, China

|  | Total (n=146) | Clustered (n=36) | Unique (n=110) | P value |
| --- | --- | --- | --- | --- |
| Sex |  |  |  | 0.029 |
| Male | 104 (71.23%) | 20 (55.56%) | 84 (76.36%) |  |
| Female | 42 (28.77%) | 16 (44.44%) | 26 (23.64%) |  |
| Age group |  |  |  | 0.124 |
| 15-24 | 15 (10.27%) | 1 (2.78%) | 14 (12.73%) |  |
| 25-34 | 40 (27.40%) | 13 (36.11%) | 27 (24.55%) |  |
| 35-44 | 24 (16.44%) | 4 (11.11%) | 20 (18.18%) |  |
| 45-54 | 19 (13.01%) | 4 (11.11%) | 15 (13.64%) |  |
| ≥55 | 38 (26.03%) | 14 (38.89%) | 24 (21.82%) |  |
| Birth in Beijing |  |  |  | 0.029 |
| No | 58 (39.73%) | 8 (22.22%) | 49 (44.55%) |  |
| Yes | 88 (60.27%) | 28 (77.77%) | 61 (55.45%) |  |
| Residence Years |  |  |  | 0.089 |
| <5 | 29 (19.86%) | 5 (13.88%) | 24 (21.82%) |  |
| 5-10 | 20 (13.70%) | 2 (5.55%) | 19 (17.27%) |  |
| ≥10 | 97 (66.44%) | 29 (80.55%) | 67 (60.91%) |  |
| Occupation |  |  |  | 0.347 |
| Farmers | 17 (11.64%) | 7 (19.44%) | 10 (9.09%) |  |
| Workers | 13 (8.90%) | 3 (8.33%) | 9 (8.18%) |  |
| Retired people | 24 (16.44%) | 6 (16.67%) | 18 (16.36%) |  |
| Unemployed | 25 (17.12%) | 8 (22.22%) | 17 (15.45%) |  |
| Wholesale and retail workers | 12 (8.22%) | 1 (2.78%) | 11 (10.00%) |  |
| Others | 55 (37.67%) | 11 (30.56%) | 45 (40.91%) |  |
| TB history |  |  |  | 0.561 |
| New case | 77 (52.74%) | 21 (58.33%) | 56 (50.91%) |  |
| Previous treatment | 69 (47.26%) | 15 (41.67%) | 54 (49.09%) |  |
| Cough |  |  |  | 0.800 |
| Yes | 87/116 (75.00%) | 23/30 (76.67%) | 64/86 (74.42%) |  |
| No | 29/116 (25.00%) | 7/30 (23.33%) | 22/86 (25.58%) |  |
| Smear status |  |  |  | 0.137 |
| Positive | 98 (67.12%) | 28 (77.78%) | 70 (63.64%) |  |
| Negative | 48 (32.88%) | 8 (22.22%) | 40 (36.36%) |  |
| Number of additional drug resistance mutations | 2 (2-3) | 2 (2-3) | 2 (1.25-3) | 0.510 |
| Putative compensatory mutation in rpoA, rpoB and rpoC | | |  | 0.705 |
| Yes | 43 (29.45%) | 12 (33.33%) | 31 (28.18%) |  |
| No | 103 (70.55%) | 24 (66.67%) | 79 (71.82%) |  |
|  |  |  |  |  |


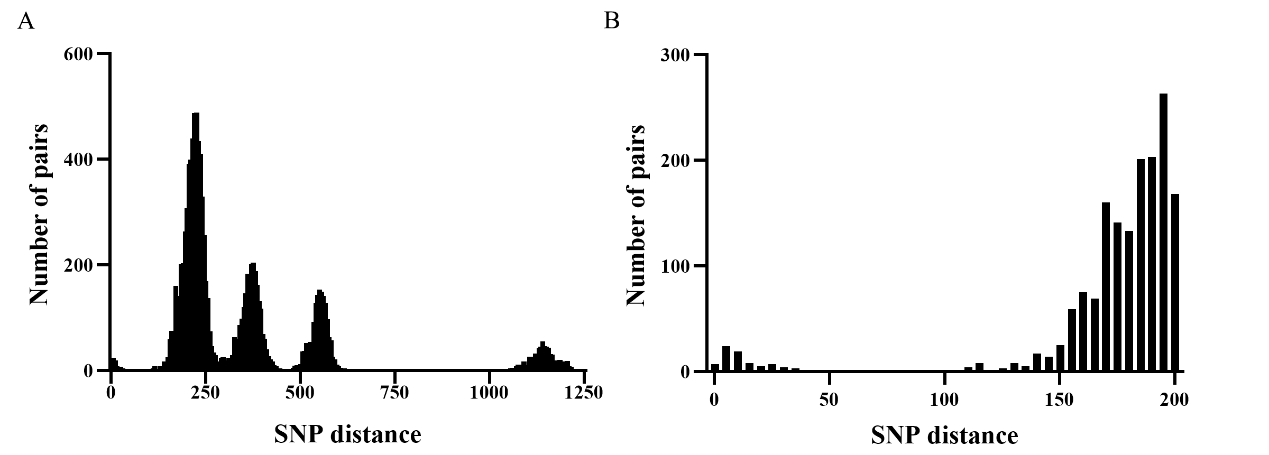


Supplement Figure 1: SNP distribution of enrolled MDR strains. (A) Total distribution of pairwise SNP distance. (B) Distribution of pairwise SNP distances≤200.


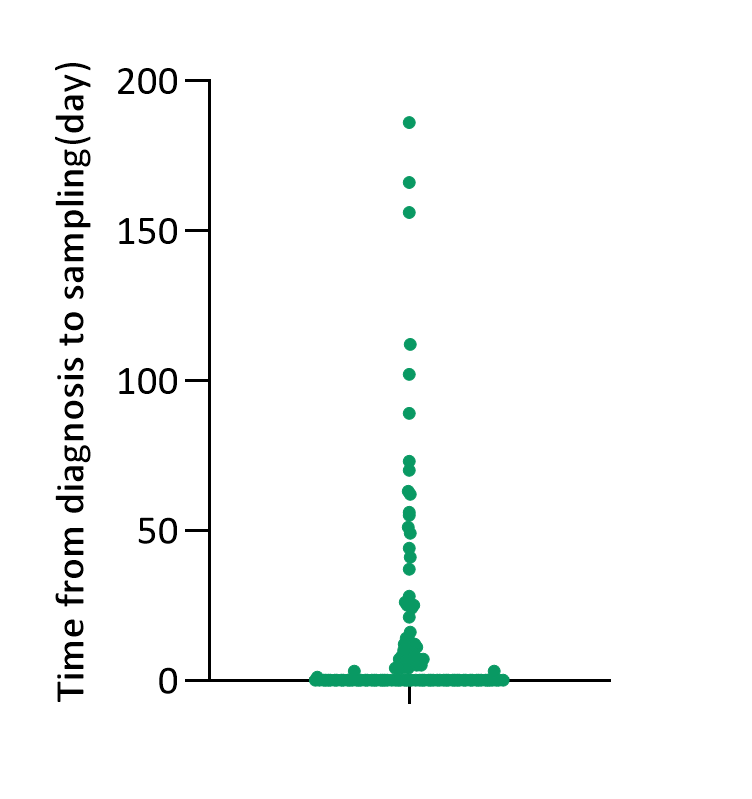


Supplement Figure 2: The distribution of time from diagnosis to sampling(day).


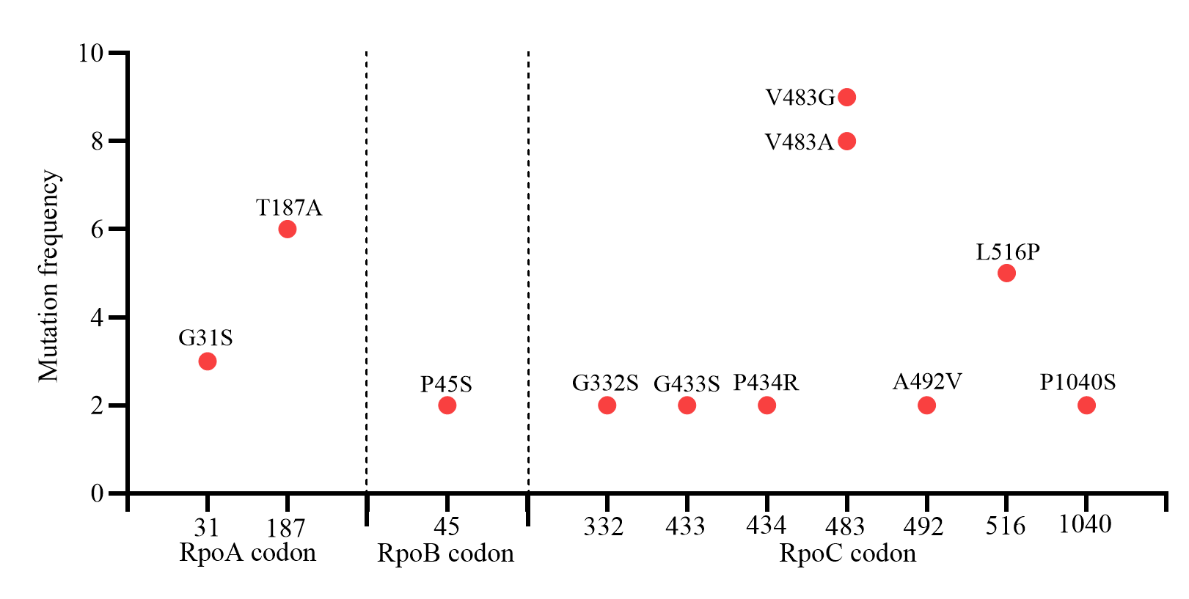


Supplement Figure 3: Putative compensatory mutations in the rpoA, rpoB, and rpoC genes identified in this study. Each putative compensatory mutation was supported by at least two independent evolution events.
